# Tallo: A global tree allometry and crown architecture database

**DOI:** 10.1111/gcb.16302

**Published:** 2022-06-28

**Authors:** Tommaso Jucker, Fabian Jörg Fischer, Jérôme Chave, David A. Coomes, John Caspersen, Arshad Ali, Grace Jopaul Loubota Panzou, Ted R. Feldpausch, Daniel Falster, Vladimir A. Usoltsev, Stephen Adu‐Bredu, Luciana F. Alves, Mohammad Aminpour, Ilondea B. Angoboy, Niels P. R. Anten, Cécile Antin, Yousef Askari, Rodrigo Muñoz, Narayanan Ayyappan, Patricia Balvanera, Lindsay Banin, Nicolas Barbier, John J. Battles, Hans Beeckman, Yannick E. Bocko, Ben Bond‐Lamberty, Frans Bongers, Samuel Bowers, Thomas Brade, Michiel van Breugel, Arthur Chantrain, Rajeev Chaudhary, Jingyu Dai, Michele Dalponte, Kangbéni Dimobe, Jean‐Christophe Domec, Jean‐Louis Doucet, Remko A. Duursma, Moisés Enríquez, Karin Y. van Ewijk, William Farfán‐Rios, Adeline Fayolle, Eric Forni, David I. Forrester, Hammad Gilani, John L. Godlee, Sylvie Gourlet‐Fleury, Matthias Haeni, Jefferson S. Hall, Jie‐Kun He, Andreas Hemp, José L. Hernández‐Stefanoni, Steven I. Higgins, Robert J. Holdaway, Kiramat Hussain, Lindsay B. Hutley, Tomoaki Ichie, Yoshiko Iida, Hai‐sheng Jiang, Puspa Raj Joshi, Hasan Kaboli, Maryam Kazempour Larsary, Tanaka Kenzo, Brian D. Kloeppel, Takashi Kohyama, Suwash Kunwar, Shem Kuyah, Jakub Kvasnica, Siliang Lin, Emily R. Lines, Hongyan Liu, Craig Lorimer, Jean‐Joël Loumeto, Yadvinder Malhi, Peter L. Marshall, Eskil Mattsson, Radim Matula, Jorge A. Meave, Sylvanus Mensah, Xiangcheng Mi, Stéphane Momo, Glenn R. Moncrieff, Francisco Mora, Sarath P. Nissanka, Kevin L. O'Hara, Steven Pearce, Raphaël Pelissier, Pablo L. Peri, Pierre Ploton, Lourens Poorter, Mohsen Javanmiri Pour, Hassan Pourbabaei, Juan Manuel Dupuy‐Rada, Sabina C. Ribeiro, Casey Ryan, Anvar Sanaei, Jennifer Sanger, Michael Schlund, Giacomo Sellan, Alexander Shenkin, Bonaventure Sonké, Frank J. Sterck, Martin Svátek, Kentaro Takagi, Anna T. Trugman, Farman Ullah, Matthew A. Vadeboncoeur, Ahmad Valipour, Mark C. Vanderwel, Alejandra G. Vovides, Weiwei Wang, Li‐Qiu Wang, Christian Wirth, Murray Woods, Wenhua Xiang, Fabiano de Aquino Ximenes, Yaozhan Xu, Toshihiro Yamada, Miguel A. Zavala

**Affiliations:** ^1^ School of Biological Sciences University of Bristol Bristol UK; ^2^ Laboratoire Évolution et Diversité Biologique (EDB) UMR 5174 (CNRS/IRD/UPS) Toulouse Cedex 9 France; ^3^ Université Toulouse Toulouse Cedex 9 France; ^4^ Conservation Research Institute University of Cambridge Cambridge UK; ^5^ Institute of Forestry and Conservation University of Toronto Toronto Ontario Canada; ^6^ Forest Ecology Research Group, College of Life Sciences Hebei University Baoding Hebei China; ^7^ Université de Liège, Gembloux Agro‐Bio Tech Gembloux Belgium; ^8^ Laboratoire de Biodiversité, de Gestion des Ecosystèmes et de l'Environnement (LBGE), Faculté des Sciences et Techniques Université Marien Ngouabi Brazzaville Republic of Congo; ^9^ College of Life and Environmental Sciences University of Exeter Exeter UK; ^10^ Evolution & Ecology Research Centre University of New South Wales Sydney Sydney New South Wales Australia; ^11^ Department of Forestry Ural State Forest Engineering University Yekaterinburg Russia; ^12^ Department of Forest Dynamics Botanical Garden of the Ural Branch of Russian Academy of Sciences Yekaterinburg Russia; ^13^ Forestry Research Institute of Ghana, Council for Scientific and Industrial Research University Kumasi Ghana; ^14^ Center for Tropical Research, Institute of the Environment and Sustainability University of California Los Angeles Los Angeles California USA; ^15^ Natural Recourses and Watershed Management Office, West Azerbaijan Province Urmia Iran; ^16^ Institut National pour l'Etude et la Recherche Agronimiques Democratic Republic of the Congo; ^17^ Center for Crop Systems Analysis Wageningen University Wageningen The Netherlands; ^18^ AMAP Lab Montpellier University, IRD, CIRAD, CNRS, INRAE Montpellier France; ^19^ Research Division of Natural Resources, Kohgiluyeh and Boyerahmad Agriculture and Natural Resources Research and Education Center, AREEO Yasouj Iran; ^20^ Departamento de Ecología y Recursos Naturales, Facultad de Ciencias Universidad Nacional Autónoma de México, Coyoacán Ciudad de México Mexico; ^21^ Forest Ecology and Forest Management Group Wageningen University Wageningen The Netherlands; ^22^ Department of Ecology French Institute of Pondicherry Puducherry India; ^23^ Instituto de Investigaciones en Ecosistemas y Sustentabilidad, Universidad Nacional Autónoma de México Morelia Michoacán Mexico; ^24^ UK Centre for Ecology and Hydrology Edinburgh UK; ^25^ University of California Berkeley Berkeley California USA; ^26^ Service of Wood Biology Royal Museum for Central Africa Tervuren Belgium; ^27^ Pacific Northwest National Laboratory Joint Global Change Research Institute College Park Maryland USA; ^28^ School of GeoSciences University of Edinburgh Edinburgh UK; ^29^ Yale‐NUS College Singapore; ^30^ ForestGEO Smithsonian Tropical Research Institute Apartado Panama Republic of Panama; ^31^ Department of Geography National University of Singapore Singapore; ^32^ Division Forest Office Ministry of Forest Dhangadhi Sudurpashchim Province Nepal; ^33^ College of Urban and Environmental Sciences and MOE Laboratory for Earth Surface Processes Peking University Beijing China; ^34^ Research and Innovation Centre, Fondazione Edmund Mach San Michele all'Adige Italy; ^35^ Institut des Sciences de l'Environnement et du Développement Rural (ISEDR) Université de Dédougou Dédougou Burkina Faso; ^36^ Bordeaux Sciences Agro‐UMR ISPA, INRAE Bordeaux France; ^37^ Nicholas School of the Environment Duke University Durham NC USA; ^38^ Shinto Labs Eindhoven The Netherlands; ^39^ Department of Geography and Planning, Queen's University Kingston Ontario Canada; ^40^ Department of Biology Washington University in St Louis St Louis Missouri USA; ^41^ CIRAD, UPR Forêts et Sociétés Montpellier France; ^42^ CSIRO Land and Water Canberra Australian Capital Territory Australia; ^43^ Institute of Space Technology, Islamabad Highway Islamabad Pakistan; ^44^ Swiss Federal Research Institute WSL Birmensdorf Switzerland; ^45^ Spatial Ecology Lab, School of Life Sciences South China Normal University Guangzhou Guangdong China; ^46^ Department of Plant Systematics University of Bayreuth Bayreuth Germany; ^47^ Centro de Investigación Científica de Yucatán A.C., Unidad de Recursos Naturales Mérida Yucatán Mexico; ^48^ Department of Botany University of Otago Dunedin New Zealand; ^49^ Landcare Research Lincoln New Zealand; ^50^ Gilgit‐Baltistan Forest Wildlife and Environment Department Gilgit Pakistan; ^51^ Research Institute for the Environment & Livelihoods Charles Darwin University Casuarina Northern Territory Australia; ^52^ Faculty of Agriculture and Marine Science Kochi University Nankoku Kochi Japan; ^53^ Forestry and Forest Products Research Institute Tsukuba Ibaraki Japan; ^54^ Institute of Forestry Tribhuvan University Hetauda Nepal; ^55^ Faculty of Desert Studies Semnan University Semnan Iran; ^56^ Department of Forestry, Faculty of Natural Resources University of Guilan Somehsara Iran; ^57^ Japan International Research Center for Agricultural Sciences Tsukuba Ibaraki Japan; ^58^ Department of Geosciences and Natural Resources Western Carolina University Cullowhee North Carolina USA; ^59^ Graduate School and Research Western Carolina Unversity Cullowhee North Carolina USA; ^60^ Faculty of Environmental Earth Science Hokkaido University Sapporo Japan; ^61^ Department of Forest Resources Management, College of Forestry Nanjing Forestry University Nanjing Jiangsu China; ^62^ Jomo Kenyatta University of Agriculture and Technology (JKUAT) Nairobi Kenya; ^63^ Department of Forest Botany, Dendrology and Geobiocoenology, Faculty of Forestry and Wood Technology Mendel University in Brno Brno Czech Republic; ^64^ Guangdong Provincial Key Laboratory of High Technology for Plant Protection, Plant Protection Research Institute Guangdong Academy of Agricultural Sciences Guangzhou Guangdong China; ^65^ Department of Geography University of Cambridge Cambridge UK; ^66^ Department of Forest and Wildlife Ecology University of Wisconsin‐Madison Madison Wisconsin USA; ^67^ Environmental Change Institute, School of Geography and the Environment University of Oxford Oxford UK; ^68^ Faculty of Forestry University of British Columbia Vancouver British Columbia Canada; ^69^ IVL Swedish Environmental Research Institute Göteborg Sweden; ^70^ Gothenburg Global Biodiversity Centre (GGBC), Gothenburg Sweden; ^71^ Faculty of Forestry and Wood Sciences Czech University of Life Sciences Prague, Prague 6 Suchdol Czech Republic; ^72^ Laboratoire de Biomathématiques et d'Estimations Forestières, Faculté des Sciences Agronomiques Université d'Abomey Calavi Cotonou Benin; ^73^ State Key Laboratory of Vegetation and Environmental Change, Institute of Botany Chinese Academy of Sciences Beijing China; ^74^ Laboratoire de Botanique systématique et d'Ecologie, Département des Sciences Biologiques, Ecole Normale Supérieure Université de Yaoundé I Yaoundé Cameroon; ^75^ Fynbos Node, South African Environmental Observation Network Claremont South Africa; ^76^ Centre for Statistics in Ecology, Environment and Conservation, Department of Statistical Sciences University of Cape Town Rondebosch South Africa; ^77^ Department of Crop Science, Faculty of Agriculture University of Peradeniya Peradeniya Sri Lanka; ^78^ The Tree Projects Hobart Tasmania Australia; ^79^ Universidad Nacional de la Patagonia Austral (UNPA) ‐ Instituto Nacional de Tecnología Agropecuaria (INTA) ‐ CONICET Río Gallegos Santa Cruz Argentina; ^80^ Natural Resources Faculty University of Tehran Karaj Iran; ^81^ Centro de Ciências Biológicas e da Natureza Universidade Federal do Acre, Campus Universitário Rio Branco Brazil; ^82^ Systematic Botany and Functional Biodiversity, Institute of Biology Leipzig University Leipzig Germany; ^83^ Department of Natural Resources, Faculty of Geo‐information Science and Earth Observation (ITC) University of Twente Enschede The Netherlands; ^84^ UMR EcoFoG, CNRS Kourou French Guiana; ^85^ Department of Natural Sciences Manchester Metropolitan University Manchester UK; ^86^ Field Science Center for Northern Biosphere Hokkaido University Horonobe Japan; ^87^ Department of Geography University of California Santa Barbara Santa Barbara California USA; ^88^ Earth Systems Research Center University of New Hampshire Durham New Hampshire USA; ^89^ Department of Forestry and The Center for Research and Development of Northern Zagros Forestry University of Kurdistan Erbil Iran; ^90^ Department of Biology University of Regina Regina Saskatchewan Canada; ^91^ School of Geographical and Earth Sciences University of Glasgow, East Quadrangle Glasgow UK; ^92^ Systematic Botany and Functional Biodiversity, Institute of Biology University of Leipzig Leipzig Germany; ^93^ German Centre for Integrative Biodiversity Research (iDiv) Halle‐Jena‐Leipzig Leipzig Germany; ^94^ Ontario Ministry of Natural Resources North Bay Ontario Canada; ^95^ Faculty of Life Science and Technology Central South University of Forestry and Technology Changsha Hunan China; ^96^ Forest Science, New South Wales Department of Primary Industries Parramatta New South Wales Australia; ^97^ State Key Laboratory of Aquatic Botany and Watershed Ecology Wuhan Botanical Garden, Chinese Academy of Sciences Wuhan China; ^98^ Center of Conservation Biology, Core Botanical Gardens Chinese Academy of Sciences Wuhan China; ^99^ Graduate School of Integrated Sciences of Life Hiroshima University Hiroshima Japan; ^100^ Forest Ecology and Restoration Group (FORECO), Departamento de Ciencias de la Vida Universidad de Alcalá Madrid Spain

**Keywords:** allometric scaling, crown radius, forest biomass stocks, forest ecology, remote sensing, stem diameter, tree height

## Abstract

Data capturing multiple axes of tree size and shape, such as a tree's stem diameter, height and crown size, underpin a wide range of ecological research—from developing and testing theory on forest structure and dynamics, to estimating forest carbon stocks and their uncertainties, and integrating remote sensing imagery into forest monitoring programmes. However, these data can be surprisingly hard to come by, particularly for certain regions of the world and for specific taxonomic groups, posing a real barrier to progress in these fields. To overcome this challenge, we developed the Tallo database, a collection of 498,838 georeferenced and taxonomically standardized records of individual trees for which stem diameter, height and/or crown radius have been measured. These data were collected at 61,856 globally distributed sites, spanning all major forested and non‐forested biomes. The majority of trees in the database are identified to species (88%), and collectively Tallo includes data for 5163 species distributed across 1453 genera and 187 plant families. The database is publicly archived under a CC‐BY 4.0 licence and can be access from: https://doi.org/10.5281/zenodo.6637599. To demonstrate its value, here we present three case studies that highlight how the Tallo database can be used to address a range of theoretical and applied questions in ecology—from testing the predictions of metabolic scaling theory, to exploring the limits of tree allometric plasticity along environmental gradients and modelling global variation in maximum attainable tree height. In doing so, we provide a key resource for field ecologists, remote sensing researchers and the modelling community working together to better understand the role that trees play in regulating the terrestrial carbon cycle.

## Graphical abstract

Trees vary enormously in the size and shape of their crowns, and accurately capturing this incredible variation in tree architecture underpins numerous lines of ecological research. To facilitate this work, we compiled the Tallo database ‐ the world's largest open access collection of trees for which stem diameter, height and crown size have been measured. Tallo includes 498,838 georeferenced records from 61,856 sites and 5,163 tree species. This new database represents a key resource for field ecologists, remote sensing researchers and the modelling community working together to better understand the role that trees play in regulating the terrestrial carbon cycle.

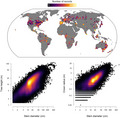

## 1 INTRODUCTION

Trees vary enormously in the size and shape of their crowns, and accurately capturing and describing this incredible variation in tree architecture is central to numerous lines of ecological research (Verbeeck et al., 2019). For instance, data capturing the relationship between the stem diameter, height and crown radius of trees have been used to test theory linking body size and metabolism across ecological scales (Coomes et al., 2012; Enquist et al., 2009; Shenkin et al., 2020), as well as exploring the ecological, environmental and evolutionary constraints that shape allometric scaling relationships of woody plants (Banin et al., 2012; Jucker et al., 2015; Lines et al., 2012; Loubota Panzou et al., 2021). These data also underpin efforts to develop more accurate and generalizable models for estimating forest biomass stocks and their uncertainties (Chave et al., 2014; Goodman et al., 2014; Jucker et al., 2017; Ploton et al., 2016). Moreover, tree height and crown size data are increasingly being used to bridge the gap between remote sensing and traditional field ecology (Aguirre‐Gutiérrez et al., 2021; Jucker et al., 2017; Marconi et al., 2021), including facilitating the integration of remote sensing data into individual‐based models of forest structure and dynamics (Fischer et al., 2019, 2020; Taubert et al., 2015). However, because basic properties of tree size, such as their height and crown dimensions, are challenging and time‐consuming to measure accurately on the ground, limited access to curated tree crown architectural data is often a real barrier to progress in these fields.

**FIGURE 1:**
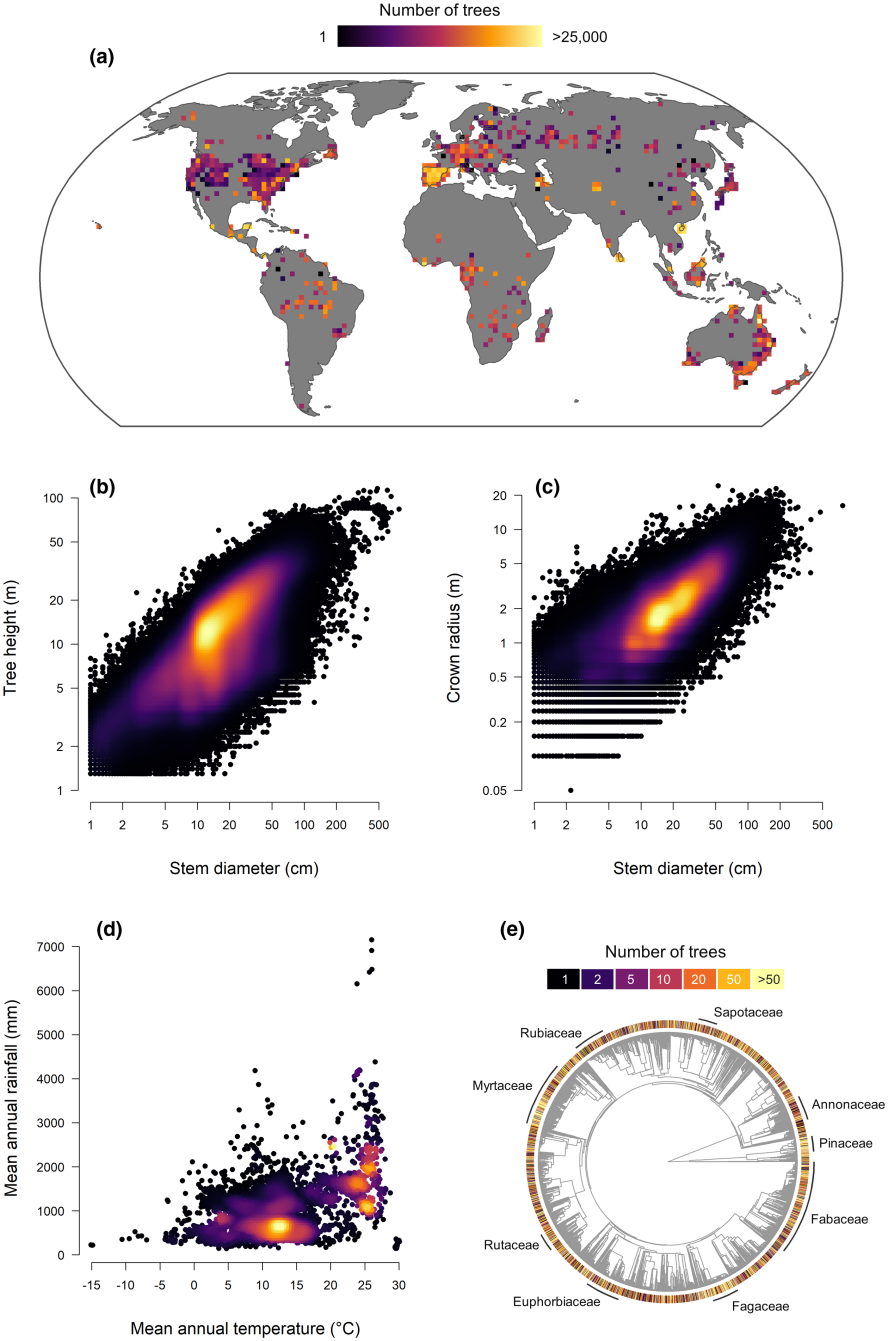
Overview of the Tallo database, including (a) geographical coverage, (b–c) size range of sampled trees, (d) climatic range of the data and (e) taxonomic coverage in phylogenetic space. Panel (a) shows the total number of trees recorded in grid cells of approximately 200 × 200 km. In (b–d), the density of overlapping points is reflected by a colour gradient ranging from black (low point density) to yellow (high point density). Data on mean annual rainfall and temperature show in (d) were obtained from WorldClim2 database (Fick & Hijmans, 2017) at a spatial resolution of 30 arc‐seconds (approximately 1 km). Panel (e) shows a phylogenetic tree constructed from all species in the Tallo database (*n* = 5163). Branch tips have been colour coded to reflect the number of trees sampled for each species and the position of several seed plant families on the tree has been labelled. The phylogenetic tree was generated using the V.PhyloMaker package in R (Jin & Qian, 2019), the backbone of which is a phylogeny of 79,881 taxa of seed plants developed by Smith and Brown (2018).

Building on previous efforts to compile regional and global tree allometry databases (Falster et al., 2015; Feldpausch et al., 2011; Jucker et al., 2017; Loubota Panzou et al., 2021), here we bring together the world's largest open access collection of trees for which stem diameter, height and/or crown radius have been measured—the Tallo database (Figure 1). Tallo includes nearly 500,000 georeferenced and taxonomically standardized records from more than 5000 tree species acquired at over 60,000 sites worldwide, including data from all major terrestrial biomes and some of the world's largest ever recorded trees. After describing the key steps involved in the acquisition and standardization of the data, we showcase some of the potential applications of the Tallo database through a series of three case studies.

## 2 COMPILING THE DATABASE

### 2.1 Data aggregation

We compiled data on trees for which stem diameter (*D*, in cm), total tree height (*H*, in m) and/or crown radius (*CR*, in m) were measured. *D* was measured at breast height (1.3 m aboveground) or otherwise just above buttress roots using either diameter tape or callipers. As is common practise, for multi‐stemmed trees a single pooled value of *D* was calculated by summing the diameter values of all individual stems (*D*_*i*_) using the quadratic diameter: $D=\sqrt{\sum D_{i}^{2}}$ (de Souza et al., 2021; Paul et al., 2016). While care was taken to identify records from multi‐stemmed individual, it is possible that for records compiled from existing databases a small number of multi‐stemmed trees were mistakenly treated as separate individuals.

*H* and *CR*—which we mostly derived from 2 to 8 orthogonal crown radii measurements or otherwise from crown projection areas—were measured using a variety of approaches, including laser or ultrasonic range finders, clinometers, as well as tape measures and telescopic poles for smaller trees. For a very small subset of trees with fully sun‐exposed crowns, *H* and *CR* were measured using a combination of high‐resolution aerial photos and airborne LiDAR (Cano et al., 2019). Previous work comparing tree height measurements derived using laser range finders and clinometers—the two most common methods used to take tree biometric measurements—has shown that the two approaches provide consistent estimates, with laser rangefinders allowing for greater precision but with a tendency to slightly underestimate total tree heights (Larjavaara & Muller‐Landau, 2013).

In addition to the data on tree size and shape, we also recorded the latitude and longitude of the site where each tree was measured, and any available taxonomic information. Data were obtained from a range of sources, including the published literature, online databases and unpublished data collected by co‐authors of this study (see Appendix S1 in Supporting Information for a complete list of data sources). In compiling these data, we excluded records from heavily managed and industrial tree plantations, as well as agroforestry systems. Care was also taken to avoid double counting trees, which could occur either because the same data had been obtained from multiple sources or because trees were measured more than once as part of successive forest inventories. Specifically, for data obtained from public databases we made sure none had been compiled from the same primary sources. Additionally, for the small subset of trees that had been measured more than once, we only retained data from the most recent census.

### 2.2 Taxonomy

Species names were cross‐referenced and harmonized against those of The Plant List (TPL; http://www.theplantlist.org), using a combination of the taxonstand package in R (Cayuela et al., 2012; R Core Development Team, 2021) and the Taxonomic Name Resolution Service (Boyle et al., 2013). While TPL has been static since 2013, it was chosen as a reference as it remains widely used in ecology. Future versions of Tallo will align to the World Flora Online (http://www.worldfloraonline.org) as this becomes the new standard for plant taxonomy. Taxa that did not match TPL were reviewed manually and any misspellings or synonyms that had not been automatically detected were corrected. The small number of species for which no direct match to TPL was found (*n* = 43, 0.8% of the total) were checked against the Global Tree Search database (GTS; https://tools.bgci.org/global_tree_search.php), a curated list of over 60,000 plant species that meet the IUCN's Global Tree Specialist Group definition of a tree. In all, 11 species (0.2% of the total) did not match either TPL or GTS, but because all of these could be traced back to published records online, we retained them in the database. Finally, genus names were used to assign each tree to its family and group trees into major divisions of vascular plants (i.e. angiosperms and gymnosperms) following the classification of Kew Royal Botanic Gardens (http://data.kew.org/vpfg1992/genfile.html).

Having standardized taxonomic names, we then removed any records from species that did not meet our working definition of trees—perennial woody seed plants with a single dominant stem, that are self‐supporting and undergo secondary growth. This included removing all ferns, palms, lianas, strangler figs, bamboos, pandans, as well as a number of shrub species that rarely exceed 2 m in height and are generally multi‐stemmed.

### 2.3 Geographical coordinates

Each tree in the database is associated with a set of geographical coordinates, recorded in decimal degrees of latitude and longitude. These range in precision between 1 and 3 decimal places (approximately 0.1–11 km at the equator), with the majority of trees geolocated with a precision of ≤1 km. To facilitate the integration of the Tallo database with other large‐scale spatial datasets, we used the CoordinateCleaner package in R to flag and correct common issues known to affect georeferenced records obtained from online databases (Zizka et al., 2019). First, we checked for any coordinates that were either invalid or had zero values for longitude or latitude. Data from two locations with a longitude of zero were retained after being checked against primary sources. Next, we checked for coordinates that did not fall on land by overlaying them onto a map of the world's coastlines obtained from the Natural Earth database at 1:10, 1:50 and 1:110 million scales (https://www.naturalearthdata.com). A small number of locations (*n* = 13 sites, 0.02% of the total) were located at sea at all three scales. These records were all from the Balearic Islands in Spain and were manually corrected to the nearest land point using the 1:10 million scale map as a reference. Lastly, we also checked that all coordinates aligned to high‐resolution (30 arc‐seconds, approximately 1 km) gridded climate data from the WorldClim2 database (Fick & Hijmans, 2017).

### 2.4 Data quality control

As a quality control measure, we first removed any trees recorded as dead or damaged and then filtered the database to exclude trees with *D* < 1 cm and *H* < 1.3 m. We then used Mahalanobis distance as implemented in the OutlierDetection package in R to identify trees with unrealistically large or small *H* and *CR* values given the size of their trunk, their biome association and their functional group (see Appendix S2 for details). These outliers could be the result of a data entry error (e.g. shift in decimal place or mistaken conversion between m, cm and mm) or possibly reflect a tree with substantial damage to its crown which went unrecorded. In total, 508 trees were identified as outliers based on *H* (Figure S1) and a further 490 based on *CR* (Figure S2). These records were retained in the Tallo database but flagged as outliers, allowing them to be easily removed by users depending on the application.

## 3 DATABASE OVERVIEW AND ACCESS

The Tallo database includes a total of 498,838 trees from 61,856 unique sites across the world for which *D*, *H* and/or *CR* have been measured (Figure 1a), including 311,326 trees where all three dimensions are recorded. These represent all major forested and non‐forested biomes (Table 1) and cover a gradient of over 45°C in mean annual temperature and more than 7000 mm in annual rainfall (Figure 1d). Trees in the database span multiple orders of magnitude in size (Figure 1b–c and Table 1), with *D* ranging from 1.0 to 770.0 cm, *H* from 1.3 to 115.8 m and *CR* from 0.05 to 24.25 m. The majority of trees (88%) are identified to species, and overall the Tallo database includes records for 5163 species, 1453 genera and 187 plant families (Figure 1e).

**TABLE 1:** Breakdown of the Tallo database by biome, including number of tree records and species, as well as the median and range of stem diameter (*D*, in cm), tree height (*H*, in m) and crown radius (*CR*, in m) values. Biome classifications follow those of Olson et al. (2001), with boreal and montane biomes grouped together

Biome	N° trees	N° species	*D* (in cm)	*H* (in m)	*CR* (in m)
Tropical rain forests	179,175	3547	13.5 [1–475.3]	12.5 [1.3–100.8]	1.5 [0.05–24.25]
Tropical dry forests	30,117	526	6.6 [1–175.0]	6.0 [1.3–65]	1.25 [0.05–21.0]
Temperate broadleaf forests	126,517	781	16.3 [1–652.0]	11.0 [1.3–99.7]	2.0 [0.05–17.5]
Temperate conifer forests	26,849	208	15.2 [1–770.0]	11.1 [1.3–115.8]	1.7 [0.05–16.25]
Boreal & montane forests	21,631	37	17.0 [1–181.0]	12.9 [1.3–76]	1.35 [0.05–5.1]
Mediterranean woodlands	80,882	140	21.4 [1–403.0]	7.5 [1.4–76.7]	2.0 [0.25–16]
Tropical savannas	22,818	587	12.9 [1–251.0]	8.7 [1.3–66.2]	1.5 [0.1–22.05]
Temperate grasslands	9572	126	11.1 [1–117.0]	8.4 [1.3–48.6]	1.2 [0.05–9.6]
Drylands	538	17	9.3 [1–40.3]	2.8 [1.3–10.7]	1.5 [0.4–4.95]
Mangroves	739	3	13.2 [1–103.3]	10.0 [1.3–32.2]	1.5 [0.2–7.4]

The version of the Tallo database described in this article is publicly archived on Zenodo under a CC‐BY 4.0 licence so that it can be freely used, shared and modified so long as appropriate credit is given (https://doi.org/10.5281/zenodo.6637599). Major version updates will be periodically uploaded to Zenodo, in addition to which we will also maintain an up‐to‐date version of the database on GitHub (https://github.com/selva‐lab‐repo/TALLO). Tallo should be referenced by citing this paper and users are also encouraged to report the version of the database they have accessed and to cite the original data sources whenever possible. The database is stored as a csv file which contains the individual tree morphological data, the geographical coordinates of each tree, any available taxonomic information, an identifier flagging any records classified as outliers and a reference code linking to the source from which records were obtained. A look‐up table with full bibliographical sources is provided separately in Table S1 and as a csv file on the GitHub repository. Additionally, metadata files with a detailed description of each field in Tallo database can also be found on GitHub.

## 4 CASE STUDIES

To showcase a few of the possible applications of the Tallo database, we developed three case studies that explore a range of theoretical and applied questions in ecology related to tree allometry. To enable users to replicate and build on these examples, all R code and ancillary environmental data used in the case studies have been archived on the GitHub repository.

### 4.1 Case study 1: Testing the predictions of metabolic scaling theory across biomes and functional groups

Metabolic scaling theory (MST) makes a number of predictions about how different axes of tree size should scale against one another based on first principles of plant water transport, branching architecture and stem biomechanics (Anderson‐Teixeira et al., 2015; Shenkin et al., 2020; West et al., 2009). Specifically, both *H* and *CR* are expected to be proportional to the 23 power of *D* (i.e. H∝D23 and CR∝D23), while *CR* is assumed to scale isometrically with *H* (i.e. $\mathrm{CR}\propto H^{1}$). However, there is substantial evidence that real‐world scaling relationships can depart substantially and systematically from the theoretical predictions of MST due to the environmental context in which a tree is growing (e.g. water availability, competition for light, browsing, disturbance regime), as well as its evolutionary history (Jucker et al., 2017; Lines et al., 2012; Moncrieff et al., 2011; Muller‐Landau et al., 2006; Shenkin et al., 2020).

Using Tallo, we tested whether crown allometric scaling relationships can be reconciled with the predictions of MST or if instead they vary systematically and predictably among major plant lineages (i.e. angiosperms and gymnosperms) and biome types. We modelled *H–D*, *CR–D* and *CR–H* scaling relationships using a power‐law function by fitting linear mixed‐effects regressions to log–log transformed data and allowing both the normalization constant (intercept) and scaling exponent (slope) to vary among biomes for both angiosperm and gymnosperm lineages. Models were fit using the lme4 package in R and took the following general form:
lmer(log(Y) ~ log(X) + (log(X)|Biome:Lineage))

Additionally, we also tested whether biome‐level scaling exponents varied in relation to the degree of aridity experienced by trees within a biome, quantified as the ratio between mean annual precipitation and potential evapotranspiration. Aridity data for this analysis were obtained at 30‐arc second resolution from the Global Aridity Index and PET Database (Trabucco & Zomer, 2019) and matched to each tree based on its geographical coordinates.

**FIGURE 2:**
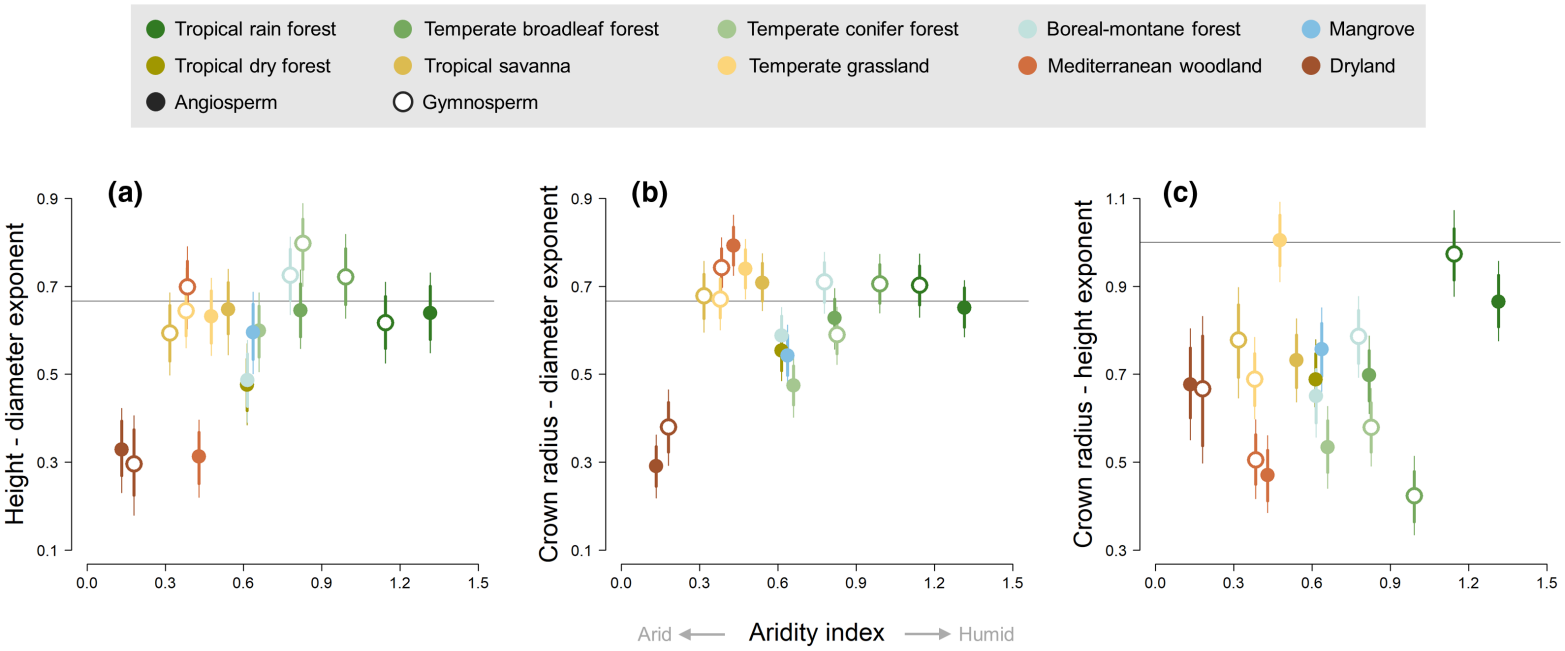
Variation in height–diameter (a), crown radius–diameter (b) and crown radius–height (c) scaling exponents of angiosperm (filled circles) and gymnosperm (empty circle) trees growing in different biome types arranged according to their aridity index. Error bars denote both the 80% (thick lines) and the 95% confidence intervals (thin lines) of the parameter estimates. Grey horizonal lines indicate scaling exponents predicted by metabolic scaling theory. Biome classification follows that of Olson et al. (2001), while aridity was calculated as the ratio between mean annual precipitation and potential evapotranspiration and therefore ranges from arid at low values of the index to humid at higher values.

We found that agreement with MST varied considerably among the two plant lineages and biomes, as well as the allometric relationship being examined (Figure 2). Overall, observed *H–D* scaling exponents were lower than the 23 predicted by MST (exponent estimate ±95% confidence intervals = 0.581 ± 0.068). This was especially true for angiosperm trees, whose *H–D* scaling exponents were on average substantially lower than those of gymnosperms across biomes (0.537 and 0.637, respectively). However, for both lineages departure from MST was most pronounced in arid biomes, whereas *H–D* scaling exponents of trees growing in non‐water limited ecosystems such as tropical and temperate forests were consistent with the predictions of MST (Figure 2a). This trend was reflected in a significantly positive correlation between a biome's *H–D* scaling exponent and the mean aridity index experience by trees with that biome (Pearson's correlation coefficient, *ρ* = 0.56, *p* = .017).

A similar picture emerged for *CR–D*, where once again observed scaling exponents were on average lower than those predicted by MST (Figure 2b), although in this case 95% confidence intervals of the parameter estimate overlapped with 23 (0.620 ± 0.062). On average, gymnosperms had higher *CR–D* scaling exponents than angiosperms (0.648 and 0.597, respectively) and trees growing in drylands had *CR–D* scaling exponents that were furthest from those predicted by MST (although in this case the correlation with aridity was not statistically significant; *ρ* = 0.35, *p* = .15). By contrast, *CR–H* scaling relationships showed a much bigger departure from MST predictions, with an overall scaling exponent well below 1 (0.695 ± 0.077). This was true for both angiosperms and gymnosperms (0.708 and 0.676 on average, respectively) and was broadly consistent across biomes (Figure 2c). In fact, while we did observe a couple of groups for which *CR–H* scaling exponents match MST predictions (e.g. angiosperm trees in the temperate grassland biome and gymnosperm trees in tropical rain forests), no clear relationship emerged between a biome's *CR–H* scaling exponent and its degree of aridity (*ρ* = 0.22, *p* = .38).

Overall, our analysis indicates that tree crown allometries only conform to MST under certain environmental conditions and tend to do so more for gymnosperms than angiosperms. Moreover, while *CR–D* relationships were found to be consistent with MST across most biomes, they did so despite clear deviations of *CR–H* relationships from which the former are derived (Shenkin et al., 2020). This suggests that while MST may serve as a useful starting point for understanding scaling relationships between different axes of tree size, at least some of its underlying assumptions need to be revisited.

### 4.2 Case study 2: Plasticity in height–diameter scaling relationships along aridity gradients

Trees adapt their size and shape to match the environment in which they grow (Jucker et al., 2015; Kafuti et al., 2022; Lines et al., 2012). A classic example is the fact that trees tend to be shorter for a given stem diameter in drier environments (Chave et al., 2014; Hulshof et al., 2015; Jucker et al., 2017; Lines et al., 2012; Vieilledent et al., 2012), as taller trees are generally at greater risk of hydraulic failure due to embolism and this effect is exacerbated when access to water becomes progressively more limiting (Domec et al., 2008; McDowell & Allen, 2015; Olson et al., 2018; Stovall et al., 2019). However, it remains unclear to what extent the negative relationship between tree height and aridity is driven by turnover in species composition along environmental gradients (i.e. species with shallower *H–D* scaling relationships dominating arid environments and vice versa) as opposed to plasticity and local adaptation within species (Lines et al., 2012).

To answer this question, we selected a subset of species in the Tallo database that had been sampled at multiple sites spanning a gradient in aridity (defined and quantified in the same way as the previous case study). Specifically, we only included records for species that (i) were found at two or more distinct sites with at least 10 individual trees sampled at each site, (ii) were recorded at locations with at least a 20% difference in aridity index between their most arid and humid site and (iii) spanned a size range of at least 20 cm in stem diameter. This left us with 155,002 trees belonging to 342 species (303 angiosperms and 39 gymnosperms). Using these data, we tested how *H–D* relationships (modelled on a log–log scale) vary along aridity gradients in relation to both species turnover and within‐species plasticity by fitting the following linear mixed‐effects model in the lme4 package in R:
lmer(log(H) ~ log(D) + AI_SP_ + AI_GMC_ + (log(D) + AI_GMC_|Species))

where AI_SP_ is the mean aridity index value of each species and AI_GMC_ is the group‐mean centred aridity index value of each tree (calculated as the difference between each tree's aridity index value and AI_SP_, the mean value of its species). The AI_SP_ term in the model tests whether tree species found in more arid environments tend to be shorter, for a given stem diameter, than those from more humid regions. Instead, AI_GMC_ tests whether individuals within a species growing at the arid‐end of at their distribution (where AI_GMC_ < 0) are shorter than those at the humid‐end (where AI_GMC_ > 0). The effects of both log(D) and AI_GMC_ on log(H) were allowed to vary among species (i.e. random intercept and slopes model) and a permutation approach was used to generate 95% confidence intervals for the random slope terms of the model. This allowed us to determine which tree species exhibited significantly negative or positive shifts in height in response to rising aridity.

**FIGURE 3:**
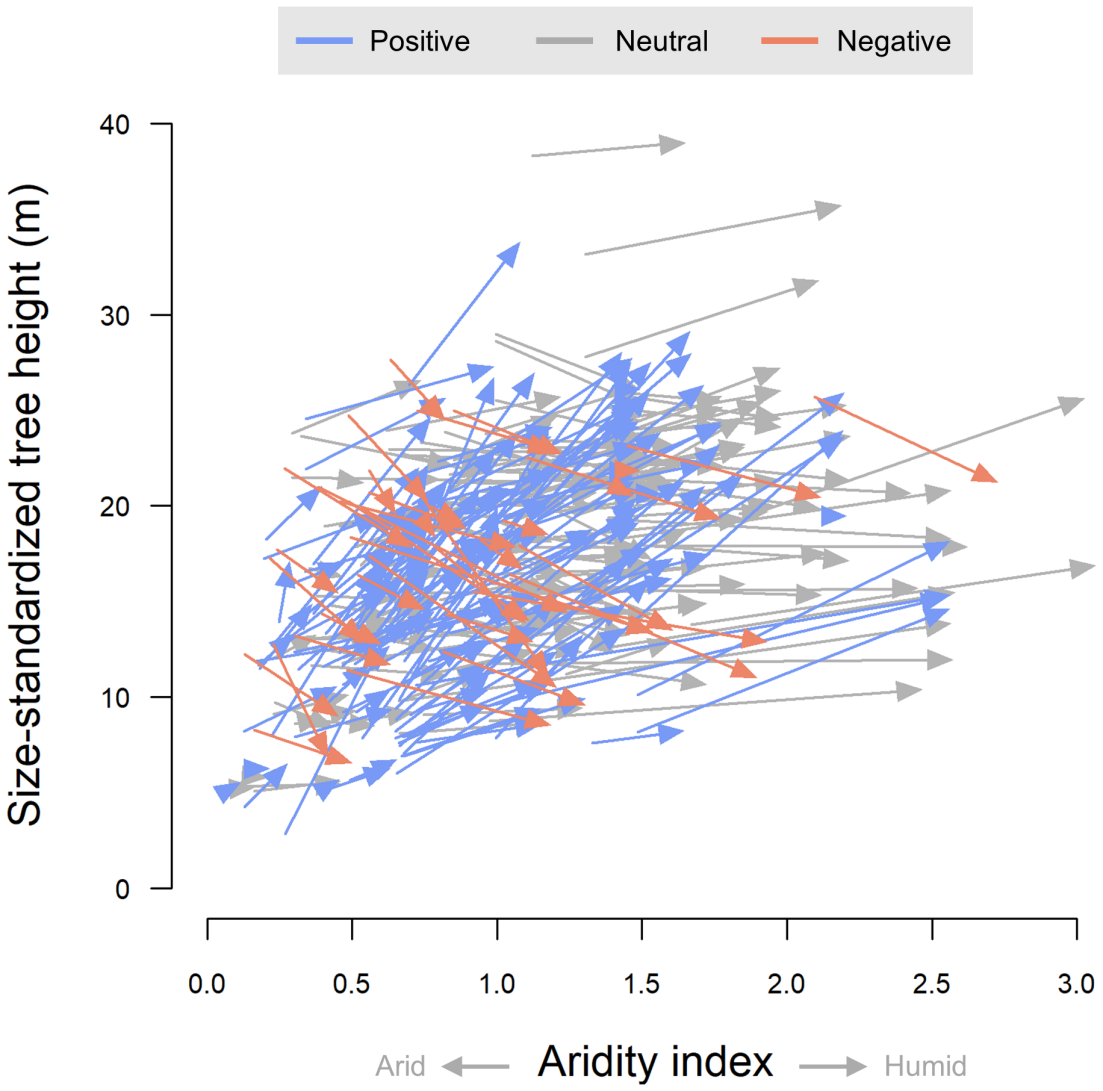
Variation in the height of a tree with a stem diameter of 30 cm (*H*_*D=30cm*_) across a gradient of aridity. Each arrow corresponds to one of 342 species, with the beginning and end of the arrow indicating the species' predicted *H*_*D=30cm*_ at the arid and humid end of its sampled distribution, respectively. Blue arrows denote species for which *H*_*D=30cm*_ increased significantly as aridity decreased (*n* = 147), while those in red showed the opposite trend (*n* = 37). Aridity was calculated as the ratio between mean annual precipitation and potential evapotranspiration and ranges from arid at low values of the index to humid at higher values.

We found that aridity plays a key role in modulating the relationship between tree height and stem diameter, with trees growing in more arid environments generally much stouter than those from more humid climates (Figure 3). For example, a 30 cm diameter tree growing in a location where mean annual rainfall is only half of potential evapotranspiration (aridity index = 0.5) is on average 9.7 m shorter (−42%) than one growing where annual rainfall is double the evaporative demand (aridity index = 2). Standardized model coefficients for AI_SP_ (0.145 ± 0.026) and AI_GMC_ (0.085 ± 0.021) were both significantly positive (*p* < .0001). This indicates that the strong effect of aridity on *H–D* scaling relationships is driven by a combination of both species turnover and intraspecific plasticity across aridity gradients, with the former playing a particularly important role.

While we found that decreasing aridity generally led to trees becoming more slender, this effect varied considerably among species. Most species (*n* = 241, 70% of the total) tended to be taller at the humid end of their sampled distribution, with 44% exhibiting a significantly positive increase in height with decreasing aridity (i.e. lower bounds of the 95% confidence intervals of the random AI_GMC_ slope > 0; blue arrows in Figure 3). However, we also observed a smaller proportion of species that exhibited the opposite trend (*n* = 38, 11% of the total; red arrows in Figure 3). In relative terms, these were more likely to be gymnosperms (26% of species) than angiosperms (9% of species). Moreover, we found that species adapted to drier environments were generally more likely to respond positively to increased water availability in terms of investment in height growth compared to those from more humid climates (*ρ* = −0.18, *p* = .0006 when relating a species' random AI_GMC_ slope estimate to its AI_SP_). Overall, our results confirm the importance of water availability in shaping *H–D* scaling relationships in trees and shed new light on the role that both species turnover and intraspecific plasticity play in driving these patterns.

### 4.3 Case study 3: Global maps of potential tree height under current and future climates

Large, tall trees play a disproportionately big role in shaping carbon cycling on land, as they store the vast majority of the aboveground biomass in a given patch of forest (Bastin et al., 2018; Lutz et al., 2018; Slik et al., 2013). Tree height is also a key axis of habitat structural complexity and plays a major role in determining habitat diversity and the buffering effect that forest canopies exert on local microclimates (Atkins et al., 2022; de Frenne et al., 2021; Jucker, Bongalov, et al., 2018; Jucker, Hardwick, et al., 2018). However, tall trees are predicted to be among the most vulnerable to climate change, as they are particularly prone to hydraulic stress (Bennett et al., 2015; McDowell & Allen, 2015; Olson et al., 2018; Stovall et al., 2019), making it critical to identify the environmental conditions under which tall trees can thrive. Most efforts to tackle this challenge have used global or regional maps of forest canopy height derived from remote sensing as a starting point and then worked backward to infer the environmental drivers that shape the distribution of tall forests (Gorgens et al., 2021; Scheffer et al., 2018; Zhang et al., 2016). But an alternative bottom‐up approach to answering this question is to build an allometric model that predicts a tree's potential height anywhere in the world based on current‐day and future environmental conditions (Chave et al., 2014).

To trial this approach, we used the entire Tallo database to fit a multiple regression model in which we expressed tree height (log(H); log‐transformed) as a function of stem diameter (log(D); log‐transformed), aridity index (log[AI]; log‐transformed), precipitation seasonality (P_SEASON_), mean annual temperature (T_MEAN_), maximum annual temperature (T_MAX_), and the interaction between AI and T_MEAN_:
lm(log(H) ~ log(D) + log(AI) + P_SEASON_ + T_MEAN_ + T_MAX_ + log(AI):T_MEAN_)

Climate data were obtained at 30‐arc second resolution and assigned to each tree based on their geographical coordinates. AI values were taken from the Global Aridity Index and PET Database, while all other climatic predictors were obtained from WorldClim2 (Fick & Hijmans, 2017). As our aim was to provide a proof of concept, climatic predictors were selected based on those identified by previous studies as playing a role in modifying *H–D* scaling relationships (Chave et al., 2014; Hulshof et al., 2015; Lines et al., 2012), rather than through an extensive model selection process.

The fitted model was used to generate spatially explicit predictions of tree height at global scale under both current and future climate scenarios at 5‐arc minute spatial resolution (approximately 10 km) obtained from WordClim2. Rather than using a single size threshold for defining ‘large trees’ across all ecosystem types, predictions were made using the 99th percentile stem diameter value of trees from each biome class in the Tallo database. These ranged from 33 cm in drylands to 95 cm in tropical rainforests. To explore how climate change might affect the distribution of tall trees, we used CMIP6 future climate projections for the period of 2061–2080 derived from the CNRM‐ESM2‐1 global climate model run under the shared socio‐economic pathway (SSP) 245 (equivalent to RCP 4.5). To simplify the analysis, stem diameter values used to define ‘large trees’ for each biome type were kept the same under both current and future climate scenarios. As such, any changes in tree height predicted by the model will result entirely from changes in *H–D* scaling relationships along climatic gradients. Given that in many ecosystems tree size distributions are predicted to shift towards smaller‐sized individuals under climate change (McDowell et al., 2020), any predicted declines in tree height may therefore be conservative. However, it is also important to note that our model predictions do not account for the effects of rising atmospheric CO_2_ on plant water use efficiency, which may offset some of the impacts of rising aridity on tree hydraulic function under global warming (Rifai et al., 2022).

**FIGURE 4:**
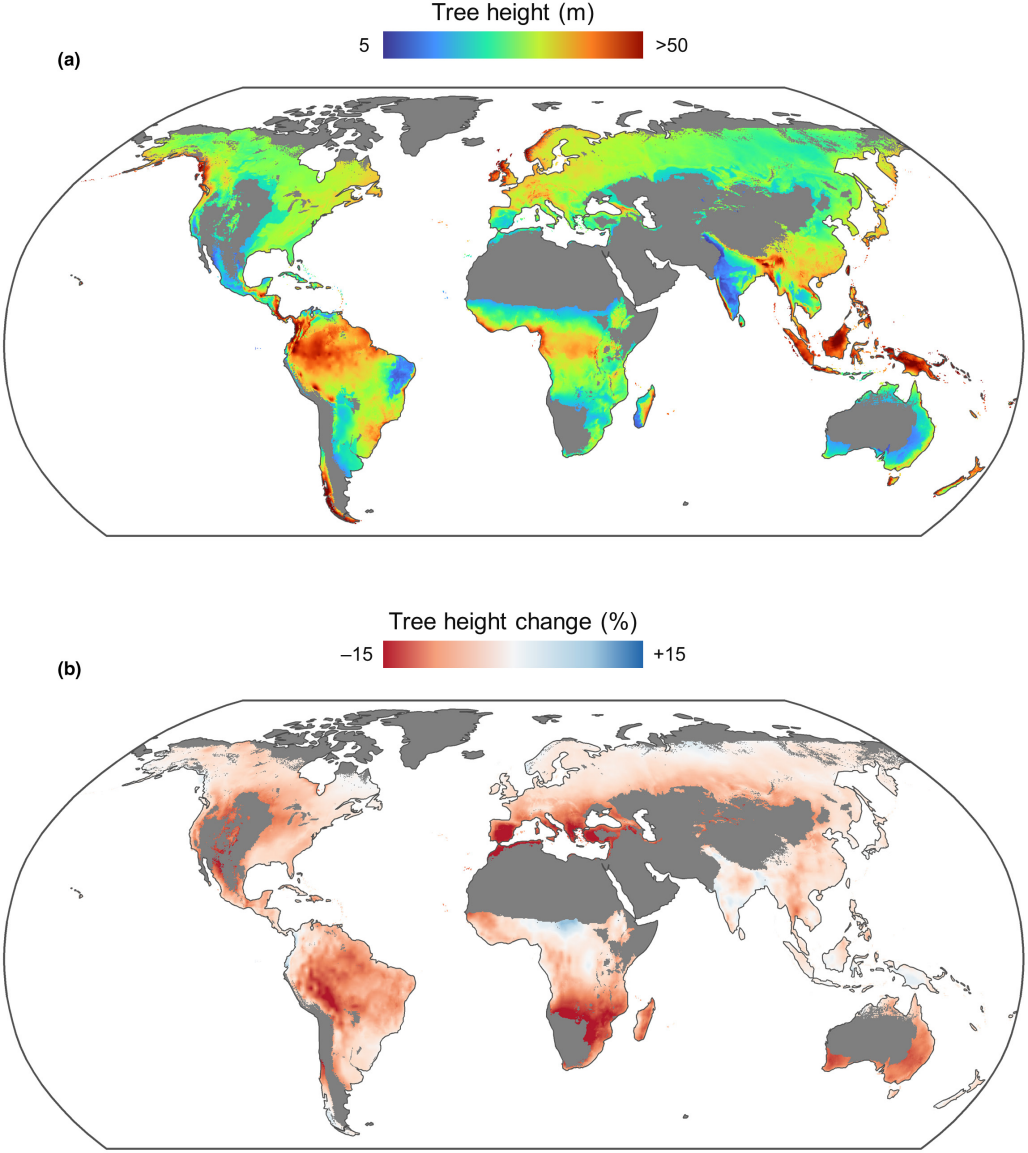
Global variation in the predicted height of large trees under current‐day climate (a) and projected relative changes in height under a future climate scenario (b). For each biome, the size threshold for ‘large trees’ was defined as the 99th percentile stem diameter value of trees in the Tallo database. Both current‐day and future climate data were obtained from the WorldClim2 database at 5‐minute resolution (Fick & Hijmans, 2017). CMIP6 future climate projections are for the period of 2061–2080 and were derived from the CNRM‐ESM2‐1 global climate model run under the shared socio‐economic pathway (SSP) 245. A map of potential forest cover (https://data.globalforestwatch.org/documents/potential‐forest‐coverage) was used to mask out areas deemed climatically unsuitable to support forests and woodlands, which are shown in dark grey.

Maps of potential tree height capture major transitions in ecosystem types (Figure 4a), with projected heights of large trees ranging between 4.7 and 69.4 m. Model predictions also capture several known hotspots of tall forests, such as those of Borneo and Southeast Asia (Banin et al., 2012; Jucker, Bongalov, et al., 2018 as well as temperate rainforests in Australia, New Zealand, the western coast of the United States, Chile and Norway (Scheffer et al., 2018). However, other regional trends in forest height are less well replicated. For instance, the map does not capture known east‐to‐west gradients in canopy height across the Amazon basin, highlighting how other drivers aside from climate—such as soils, wind, fire and herbivory—can play a key role in shaping geographical variation in forest vertical structure (Gorgens et al., 2021; Jucker, Bongalov, et al., 2018; Moncrieff et al., 2011).

In terms of projected changes in tree height in response to climate change, the height of large‐diameter trees is expected to decrease by an average of 5.4% globally when CO_2_ fertilization is not taken into account. However, projected changes varied substantially among biomes and biogeographical regions (Figure 4b), ranging anywhere between −20.1% and +18.8%. Trees in Mediterranean woodlands are predicted to show the strongest decreases in height, with an average projected height loss of 12.5%. Tropical rain forest trees are also expected to decrease in height by 5.6% on average, but this trend is much more pronounced across Amazonia and the Neotropics (−7.9%) and Africa (−4.9%) compared to Southeast Asia (−3.0%) and Australasia (−0.8%). By contrast, high‐latitude forests in the northern hemisphere are predicted to increase in height as the climate warms (Figure 4b). Overall, the Tallo database provides a new way to explore how global patterns of forest canopy structure can be reconciled with the processes that constrain the allometry of individual trees. For instance, model predictions could be compared to canopy height maps derived from remote sensing to identify areas of agreement and discrepancy between the two, providing new clues on the processes that shape variation in tree height across the world's forests. Moreover, spatially explicit maps of potential tree height generated in this way could also be used to benchmark the outputs of dynamic global vegetation models.

## 5 FUTURE DEVELOPMENTS

Looking ahead, we intend to continue curating and expanding the scope and scale of the Tallo database. In addition to increasing the geographical and taxonomic coverage of the database, we also plan to source new data capturing additional axes of crown size. In particular, we aim to incorporate data on crown depth for as many trees as possible. In addition to being interesting in its own right (Shenkin et al., 2020; Vermeulen, 2014), information on crown depth would allow users to calculate more realistic estimates of crown surface area and volume (Jucker et al., 2015; Loubota Panzou et al., 2021; Shenkin et al., 2020). Additionally, we also plan to augment the database by adding information on local competitive environment (e.g. stand basal area, tree density or cover), as it is well known that tree crown architecture is strongly influenced by competition for light with neighbouring trees (Jucker et al., 2015; Lines et al., 2012; Purves et al., 2007). As part of these efforts, we will also look to expand Tallo beyond its initial focus on seed plants with a single self‐supporting dominant stem that undergoes secondary growth—better capturing multi‐stemmed trees, as well as other life forms such as shrubs, tree ferns, palms and climbers.

Finally, as the database expands, we also plan to begin incorporating more data on crown dimensions and tree height derived from remote sensing platforms such as airborne and terrestrial laser scanning and structure‐from‐motion UAV photogrammetry. These emerging technologies allow for much more accurate and comprehensive measurements of different crown attributes (Disney, 2019), as well as capturing data on the crown dimensions of large, canopy dominant trees which tend to be disproportionately underrepresented in traditional field‐based surveys (Fischer et al., 2019; Marconi et al., 2021). To this end, we strongly encourage users to help us improve the Tallo database by not only reporting any errors they may come across, but also contributing their own data to future releases.

## AUTHORS’ CONTRIBUTIONS

T.J. conceived the idea for the Tallo database and led the aggregation of the data with the assistance of J.Ch., D.A.C., J.Ca., A.A., G.J.L.P., T.R.F., D.F. and V.A.U. and all co‐authors contributed to the data. T.J. performed the analyses with the assistance of F.J.F. T.J. wrote the first draft of the manuscript, with all authors providing editorial input.

## Supporting information


Appendix S1
Appendix S2

## Data Availability

The version of the Tallo database described in this paper is permanently archived on Zenodo (https://doi.org/10.5281/zenodo.6637599) and an updated version of Tallo is also maintained on GitHub (https://github.com/selva‐lab‐repo/TALLO). Both repositories contain a metadata file describing each field of the Tallo database and a look‐up table with the full list of bibliographical sources from which records were obtained. R code and ancillary data needed to replicate the three case studies presented in this paper can be found on the GitHub repository.
