# Supplementary material for: Tallo: A global tree allometry and crown architecture database
Source: Glob Chang Biol. 2022 Jun 28;28(17):5254–68. doi: 10.1111/gcb.16302 (PMC9542605; doi:10.1111/gcb.16302)
Supplement: Supplementary file 1 — Appendix S1 Appendix S2 [file GCB-28-5254-s001.docx]

**Supporting information**

[Appendix S1 | Sources of allometric data 2](#_Toc84841656)

[Appendix S2 | Outlier detection 4](#_Toc84841657)

[References 6](#_Toc84841658)

## Appendix S1 | Primary data sources

**Table S1** | Sources from which allometric data were compiled and corresponding reference codes in the Tallo database. Full citations for published articles are provided in the references section below.

| **Reference ID** | **Publication, database or other source** |
| --- | --- |
| 1 | Aakala et al. (2016) |
| 2 | Ali et al. (2019) |
| 3 | Alves & Santos (2002) |
| 4 | Anderson-Teixeira et al. (2015) |
| 5 | Antin et al. (2013) |
| 6 | Bongers et al. (1988) |
| 7 | Bradford et al. (2014) |
| 8 | Cano et al. (2019) |
| 9 | Caspersen et al. (2011) |
| 10 | Chave et al. (2014) |
| 11 | Cole & Lorimer (1994) |
| 12 | Coomes et al. (2014) |
| 13 | Coomes et al. (2017) |
| 14 | Dai et al. (2020) |
| 15 | Dalponte & Coomes (2016) |
| 16 | Evans et al. (2015) |
| 17 | Falster et al. (2015) |
| 18 | Fayolle et al. (2016) |
| 19 | Goodman et al. (2014) |
| 20 | Gorgens et al. (2019) |
| 21 | Groot & Luther (2015) |
| 22 | Guisasola et al. (2015) |
| 23 | Hemp et al. (2017) |
| 24 | Henry et al. (2010) |
| 25 | Henry et al. (2013) |
| 26 | Hernández-Stefanoni et al. (2014) |
| 27 | Heym et al. (2017) |
| 28 | Hickey et al. (2000) |
| 29 | Iida et al. (2012) |
| 30 | Jucker et al. (2015) |
| 31 | Jucker et al. (2016) |
| 32 | Jucker et al. (2017) |
| 33 | Jucker et al. (2022) |
| 34 | Kuyah et al. (2016) |
| 35 | Lines et al. (2012) |
| 36 | Liu et al. (2016) |
| 37 | Loubota Panzou et al. (2018) |
| 38 | Loubota Panzou et al. (2021) |
| 39 | Mifsud (2003) |
| 40 | Milodowski et al. (2021) |
| 41 | Moncrieff et al. (2014) |
| 42 | Mora et al. (2015) |
| 43 | Paul et al. (2016) |
| 44 | Ploton et al. (2016) |
| 45 | Poorter et al. (2003) |
| 46 | Poorter et al. (2006) |
| 47 | Schepaschenko et al. (2017) |
| 48 | Schlund et al. (2016) |
| 49 | Sellan et al. (2017) |
| 50 | Sellan et al. (2019) |
| 51 | Shenkin et al. (2019) |
| 52 | Shenkin et al. (2020) |
| 53 | Sterck et al. (2001) |
| 54 | Stillhard et al. (2019) |
| 55 | Sullivan et al. (2018) |
| 56 | Vovides et al. (2018) |
| 57 | Wirth et al. (2004) |
| 58 | Xu et al. (2015) |
| 59 | Australian Individual Tree Biomass Library. Available from: http://doi.org/10.4227/05/57354015127B8 |
| 60 | Central African Plot Network. Available from: https://central-african-plot-network.netlify.app |
| 61 | CMS: Forest Inventory and Biophysical Measurements, Para, Brazil, 2012-2014. Available from: https://doi.org/10.3334/ORNLDAAC/1301 |
| 62 | Forest Inventory and Analysis (FIA) – Forest Health Monitoring (FHM) program. Available from: https://www.fia.fs.fed.us/tools-data/other_data/index.php |
| 63 | Forest structure and biomass data, La Selva, Costa Rica, 2006. Available from: http://dx.doi.org/10.3334/ORNLDAAC/1215 |
| 64 | List of superlative trees. Available from: https://en.wikipedia.org/wiki/List_of_superlative_trees |
| 65 | National Ecological Observatory Network (NEON). Woody plant vegetation structure (DP1.10098.001). Available from: https://data.neonscience.org |
| 66 | Sample tree biomass data for Eurasian forests. Available from: https://elar.usfeu.ru/handle/123456789/4931 |
| 67 | Sanasilva forest health inventory. Available from: https://www.wsl.ch/de/wald/waldentwicklung-und-monitoring/sanasilva-inventur.html |
| 68 | Terrestrial Ecosystem Research Network (TERN) AusCover. Tree structural characteristics. Available from: http://www.auscover.org.au/purl/tree-structural-characteristics-all-sites. |
| 69 | The Tree Projects. Available from: https://www.thetreeprojects.com |

## Appendix S2 | Outlier detection

We used Mahalanobis distance to identify trees where tree height (*H*) and crown radius (*CR*) were unrealistically large or small given the size of their trunk, suggesting either a data entry error or possibly a tree with substantial damage to its crown. Mahalanobis distance measures the distance between a point and a distribution in bivariate space (e.g., the distribution of points in 2D space obtained when plotting *H* or *CR* against tree diameter (*D*)). Points that fall further than a certain distance from a specified percentile of the distribution are considered as outliers. Mahalanobis distance was calculated using the *maha* function in the *OutlierDetection* package in R after having log-transformed *H*, *CR* and *D* to normalise their distribution. Because *H*–*D* and *CR*–*D* relationships can vary considerably with climate and among functional groups, Mahalanobis distance was calculated separately for angiosperm and gymnosperm trees within different biomes. Trees with no taxonomic information were grouped with angiosperms, as 92% of these were from tropical forests and savannas where gymnosperms are rare. Results were checked manually and a small number of large trees that had been classed as outliers were retained in the database, as these trees departed from the main distribution of the 2D point cloud purely by virtue of being rare. In total, 508 trees were flagged as outliers based on *H* (Fig. S1) and a further 490 based on *CR* (Fig. S2).


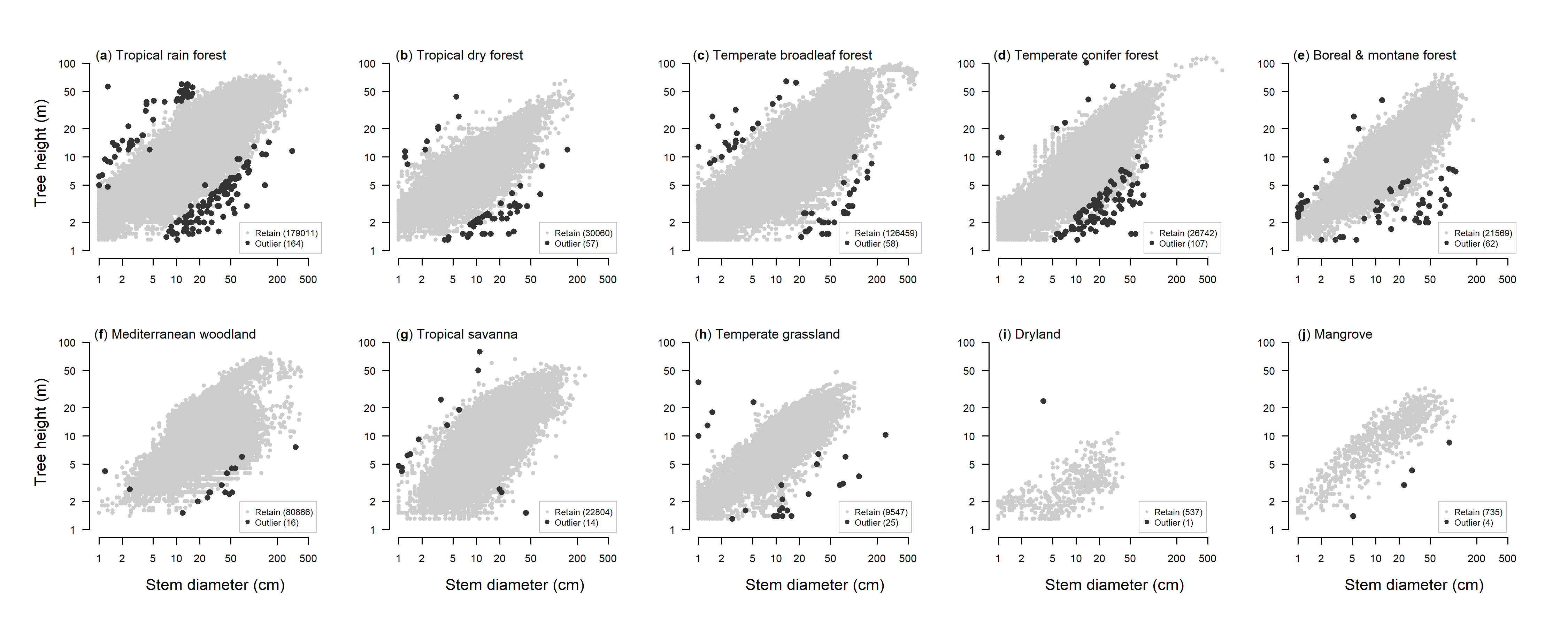


**Fig. S1 |** Trees identified as outliers based on their height (black circles) in each biome. Biome classifications follow those of Olson et al. (2001), with boreal and montane biomes grouped together. Non-outlier trees are shown for reference in light grey.


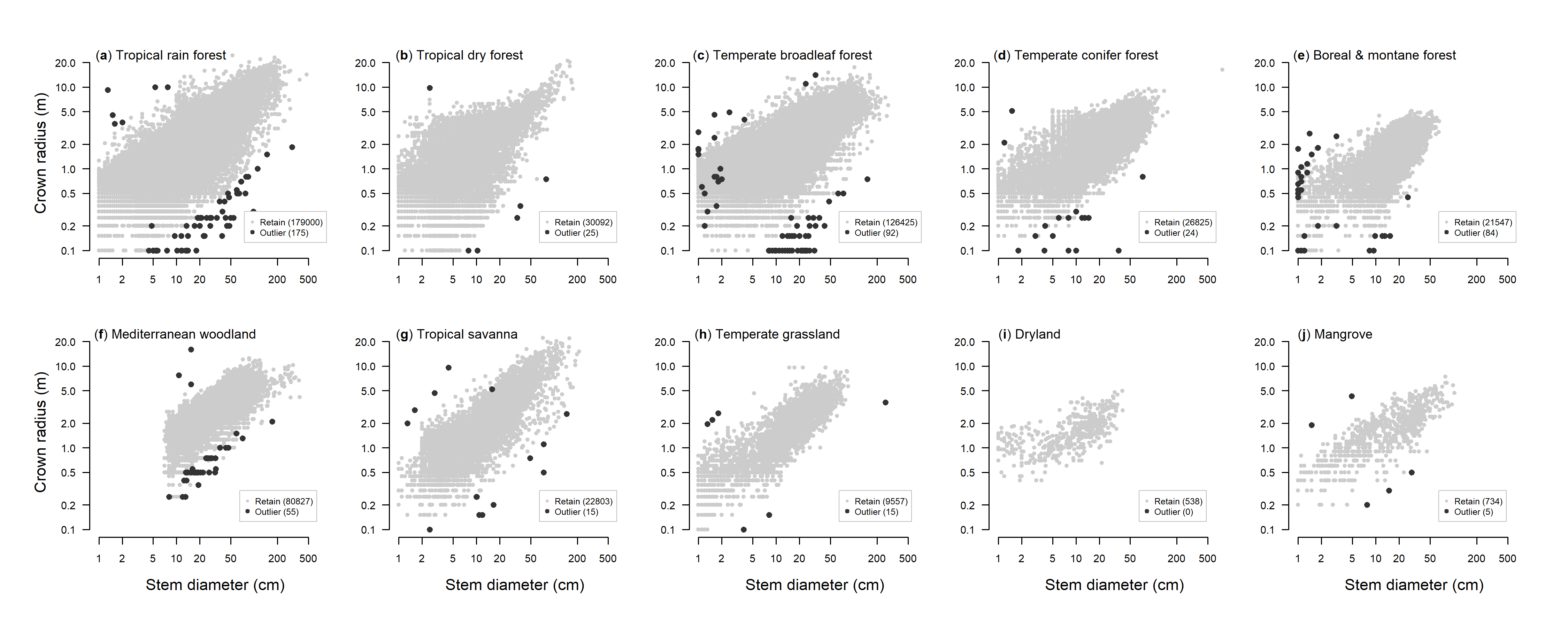


**Fig. S2 |** Trees identified as outliers based on their crown radius (black circles) in each biome. Biome classifications follow those of Olson et al. (2001), with boreal and montane biomes grouped together. Non-outlier trees are shown for reference in light grey.

## References

Aakala, T., Shimatani, K., Abe, T., Kubota, Y., & Kuuluvainen, T. (2016). Crown asymmetry in high latitude forests: disentangling the directional effects of tree competition and solar radiation. *Oikos*, *125*(7), 1035–1043. https://doi.org/10.1111/oik.02858

Ali, A., Lin, S. L., He, J. K., Kong, F. M., Yu, J. H., & Jiang, H. S. (2019). Tree crown complementarity links positive functional diversity and aboveground biomass along large-scale ecological gradients in tropical forests. *Science of the Total Environment*, *656*, 45–54. https://doi.org/10.1016/j.scitotenv.2018.11.342

Alves, L. F., & Santos, F. A. M. (2002). Tree allometry and crown shape of four tree species in Atlantic rain forest, south-east Brazil. *Journal of Tropical Ecology*, *18*(2), 245–260. https://doi.org/10.1017/S026646740200216X

Anderson-Teixeira, K. J., McGarvey, J. C., Muller-Landau, H. C., Park, J. Y., Gonzalez-Akre, E. B., Herrmann, V., Bennett, A. C., So, C. V, Bourg, N. A., Thompson, J. R., McMahon, S. M., & McShea, W. J. (2015). Size-related scaling of tree form and function in a mixed-age forest. *Functional Ecology*, *29*, 1587–1602. https://doi.org/10.1111/1365-2435.12470

Antin, C., Pélissier, R., Vincent, G., & Couteron, P. (2013). Crown allometries are less responsive than stem allometry to tree size and habitat variations in an Indian monsoon forest. *Trees - Structure and Function*, *27*(5), 1485–1495. https://doi.org/10.1007/s00468-013-0896-7

Bongers, F., Popma, J., Meave del Castillo, J., & Carabias, J. (1988). Structure and floristic composition of the lowland rain forest of Los Tuxlas, Mexico. *Vegetatio*, *74*(1), 55–80.

Bradford, M. G., Murphy, H. T., Ford, A. J., Hogan, D. L., & Metcalfe, D. J. (2014). Long-term stem inventory data from tropical rain forest plots in Australia. *Ecology*, *95*(8), 2362–000. https://doi.org/10.1890/14-0458R.1

Cano, I. M., Muller-Landau, H. C., Joseph Wright, S., Bohlman, S. A., & Pacala, S. W. (2019). Tropical tree height and crown allometries for the Barro Colorado Nature Monument, Panama: A comparison of alternative hierarchical models incorporating interspecific variation in relation to life history traits. *Biogeosciences*, *16*(4), 847–862. https://doi.org/10.5194/bg-16-847-2019

Caspersen, J. P., Vanderwel, M. C., Cole, W. G., & Purves, D. W. (2011). How stand productivity results from size- and competition-dependent growth and mortality. *PLoS ONE*, *6*(12), e28660. https://doi.org/10.1371/journal.pone.0028660

Chave, J., Réjou-Méchain, M., Búrquez, A., Chidumayo, E., Colgan, M. S., Delitti, W. B. C., Duque, A., Eid, T., Fearnside, P. M., Goodman, R. C., Henry, M., Martínez-Yrízar, A., Mugasha, W. A., Muller-Landau, H. C., Mencuccini, M., Nelson, B. W., Ngomanda, A., Nogueira, E. M., Ortiz-Malavassi, E., … Vieilledent, G. (2014). Improved allometric models to estimate the aboveground biomass of tropical trees. *Global Change Biology*, *20*, 3177–3190. https://doi.org/10.1111/gcb.12629

Cole, W. G., & Lorimer, C. G. (1994). Predicting tree growth from crown variables in managed northern hardwood stands. *Forest Ecology and Management*, *67*(1–3), 159–175. https://doi.org/10.1016/0378-1127(94)90014-0

Coomes, D. A., Dalponte, M., Jucker, T., Asner, G. P., Banin, Lindsay. F., Burslem, D. F. R. P., Lewis, S. L., Nilus, R., Phillips, O. L., Phuag, M.-H., Qiee, L., Phua, M.-H., & Qie, L. (2017). Area-based vs tree-centric approaches to mapping forest carbon in Southeast Asian forests with airborne laser scanning data. *Remote Sensing of Environment*, *194*, 77–88. https://doi.org/10.1016/j.rse.2017.03.017

Coomes, D. A., Flores, O., Holdaway, R., Jucker, T., Lines, E. R., & Vanderwel, M. C. (2014). Wood production response to climate change will depend critically on forest composition and structure. *Global Change Biology*, *20*(12), 3632–3645. https://doi.org/10.1111/gcb.12622

Dai, J., Liu, H., Wang, Y., Guo, Q., Hu, T., Quine, T., Green, S., Hartmann, H., Xu, C., Liu, X., & Jiang, Z. (2020). Drought-modulated allometric patterns of trees in semi-arid forests. *Communications Biology*, *3*(1), 1–8. https://doi.org/10.1038/s42003-020-01144-4

Dalponte, M., & Coomes, D. A. (2016). Tree-centric mapping of forest carbon density from airborne laser scanning and hyperspectral data. *Methods in Ecology and Evolution*, *10*, 1236–1245. https://doi.org/10.1111/2041-210X.12575

Evans, M. R., Moustakas, A., Carey, G., Malhi, Y., Butt, N., Benham, S., Pallett, D., & Schäfer, S. (2015). Allometry and growth of eight tree taxa in United Kingdom woodlands. *Scientific Data*, *2*(1), 1–9. https://doi.org/10.1038/sdata.2015.6

Falster, D. S., Duursma, R. A., Ishihara, M. I., Barneche, D. R., Fitzjohn, R. G., Vårhammar, A., Aiba, M., Ando, M., Anten, N., Aspinwall, M. J., Jennifer, L., Baraloto, C., Battaglia, M., Battles, J. J., Bond-lamberty, B., Van, M., Camac, J., Claveau, Y., Coll, L., … York, R. A. (2015). BAAD: a biomass and allometry database for woody plants. *Ecology*, *96*, 1445.

Fayolle, A., Loubota Panzou, G. J., Drouet, T., Swaine, M. D., Bauwens, S., Vleminckx, J., Biwole, A., Lejeune, P., & Doucet, J. L. (2016). Taller trees, denser stands and greater biomass in semi-deciduous than in evergreen lowland central African forests. *Forest Ecology and Management*, *374*, 42–50. https://doi.org/10.1016/j.foreco.2016.04.033

Goodman, R. C., Phillips, O. L., & Baker, T. R. (2014). The importance of crown dimensions to improve tropical tree biomass estimates. *Ecological Applications*, *24*(4), 680–689.

Gorgens, E. B., Motta, A. Z., Assis, M., Nunes, M. H., Jackson, T., Coomes, D., Rosette, J., Aragão, L. E. O. e C., & Ometto, J. P. (2019). The giant trees of the Amazon basin. *Frontiers in Ecology and the Environment*, *17*(7), 373–374. https://doi.org/10.1002/fee.2085

Groot, A., & Luther, J. E. (2015). Hierarchical analysis of black spruce and balsam fir wood density in Newfoundland. *Canadian Journal of Forest Research*, *45*, 805–816.

Guisasola, R., Tang, X., Bauhus, J., & Forrester, D. I. (2015). Intra- and inter-specific differences in crown architecture in Chinese subtropical mixed-species forests. *Forest Ecology and Management*, *353*, 164–172. https://doi.org/10.1016/j.foreco.2015.05.029

Hemp, A., Zimmermann, R., Remmele, S., Pommer, U., Berauer, B., Hemp, C., & Fischer, M. (2017). Africa’s highest mountain harbours Africa’s tallest trees. *Biodiversity and Conservation*, *26*(1), 103–113. https://doi.org/10.1007/s10531-016-1226-3

Henry, M., Besnard, A., Asante, W. A. A., Eshun, J., Adu-Bredu, S., Valentini, R., Bernoux, M., & Saint-André, L. (2010). Wood density, phytomass variations within and among trees, and allometric equations in a tropical rainforest of Africa. *Forest Ecology and Management*, *260*(8), 1375–1388. https://doi.org/10.1016/j.foreco.2010.07.040

Henry, M., Bombelli, A., Trotta, C., Alessandrini, A., Birigazzi, L., Sola, G., Vieilledent, G., Santenoise, P., Longuetaud, F., Valentini, R., Picard, N., & Saint-André, L. (2013). GlobAllomeTree: International platform for tree allometric equations to support volume, biomass and carbon assessment. *IForest - Biogeosciences and Forestry*, *6*(6), 326–330. https://doi.org/10.3832/ifor0901-006

Hernández-Stefanoni, J., Dupuy, J., Johnson, K., Birdsey, R., Tun-Dzul, F., Peduzzi, A., Caamal-Sosa, J., Sánchez-Santos, G., & López-Merlín, D. (2014). Improving species diversity and biomass estimates of tropical dry forests using airborne LiDAR. *Remote Sensing*, *6*(6), 4741–4763. https://doi.org/10.3390/rs6064741

Heym, M., Ruíz-Peinado, R., Del Río, M., Bielak, K., Forrester, D. I., Dirnberger, G., Barbeito, I., Brazaitis, G., Ruškytkė, I., Coll, L., Fabrika, M., Drössler, L., Löf, M., Sterba, H., Hurt, V., Kurylyak, V., Lombardi, F., Stojanović, D., Den Ouden, J., … Pretzsch, H. (2017). EuMIXFOR empirical forest mensuration and ring width data from pure and mixed stands of Scots pine (Pinus sylvestris L.) and European beech (Fagus sylvatica L.) through Europe. *Annals of Forest Science*, *74*(3), 1–9. https://doi.org/10.1007/s13595-017-0660-z

Hickey, J. E., Kostoglou, P., & Sargison, G. J. (2000). Tasmania’s tallest trees. *Tasforests*, *12*, 105–122.

Iida, Y., Poorter, L., Sterck, F. J., Kassim, A. R., Kubo, T., Potts, M. D., & Kohyama, T. S. (2012). Wood density explains architectural differentiation across 145 co-occurring tropical tree species. *Functional Ecology*, *26*(1), 274–282. https://doi.org/10.1111/j.1365-2435.2011.01921.x

Jucker, T., Bouriaud, O., & Coomes, D. A. (2015). Crown plasticity enables trees to optimize canopy packing in mixed-species forests. *Functional Ecology*, *29*(8), 1078–1086. https://doi.org/10.1111/1365-2435.12428

Jucker, T., Caspersen, J., Chave, J., Antin, C., Barbier, N., Bongers, F., Dalponte, M., van Ewijk, K. Y., Forrester, D. I., Haeni, M., Higgins, S. I., Holdaway, R. J., Iida, Y., Lorimer, C., Marshall, P. L., Momo, S., Moncrieff, G. R., Ploton, P., Poorter, L., … Coomes, D. A. (2017). Allometric equations for integrating remote sensing imagery into forest monitoring programs. *Global Change Biology*, *23*(1), 177–190. https://doi.org/10.1111/gcb.13388

Jucker, T., Fischer, J. F., Chave, J., Coomes, A. D., Caspersen, J., Ali, A., Panzou, J. L. G., Feldpausch, R. T., Falster, D., Usoltsev, A. V., Adu-Bredu, S., Alves, F. L., Aminpour, M., Angoboy, B. I., Anten, P. R. N., Antin, C., Askari, Y., Avilés, M. R., Ayyappan, N., … Zavala, A. M. (2022). Tallo – a global tree allometry and crown architecture database. *Global Change Biology*.

Jucker, T., Sanchez, A. C., Lindsell, J. A., Allen, H. D., Amable, G. S., & Coomes, D. A. (2016). Drivers of aboveground wood production in a lowland tropical forest of West Africa: teasing apart the roles of tree density, tree diversity, soil phosphorus, and historical logging. *Ecology and Evolution*, *6*(12), 4004–4017. https://doi.org/10.1002/ece3.2175

Kuyah, S., Sileshi, G., & Rosenstock, T. (2016). Allometric models based on Bayesian frameworks give better estimates of aboveground biomass in the Miombo Woodlands. *Forests*, *7*(2), 13. https://doi.org/10.3390/f7020013

Lines, E. R., Zavala, M. A., Purves, D. W., & Coomes, D. A. (2012). Predictable changes in aboveground allometry of trees along gradients of temperature, aridity and competition. *Global Ecology and Biogeography*, *21*(10), 1017–1028. https://doi.org/10.1111/j.1466-8238.2011.00746.x

Liu, X., Swenson, N. G., Lin, D., Mi, X., Umaña, M. N., Schmid, B., & Ma, K. (2016). Linking individual-level functional traits to tree growth in a subtropical forest. *Ecology*, *97*(9), 2396–2405. https://doi.org/10.1002/ecy.1445

Loubota Panzou, G. J., Fayolle, A., Feldpausch, T. R., Ligot, G., Doucet, J. L., Forni, E., Zombo, I., Mazengue, M., Loumeto, J. J., & Gourlet-Fleury, S. (2018). What controls local-scale aboveground biomass variation in central Africa? Testing structural, composition and architectural attributes. *Forest Ecology and Management*, *429*, 570–578. https://doi.org/10.1016/j.foreco.2018.07.056

Loubota Panzou, G. J., Fayolle, A., Jucker, T., Phillips, O. L., Bohlman, S., Banin, L. F., Lewis, S. L., Affum-Baffoe, K., Alves, L. F., Antin, C., Arets, E., Arroyo, L., Baker, T. R., Barbier, N., Beeckman, H., Berger, U., Bocko, Y. E., Bongers, F., Bowers, S., … Feldpausch, T. R. (2021). Pantropical variability in tree crown allometry. *Global Ecology and Biogeography*, *30*(2), 459–475. https://doi.org/10.1111/geb.13231

Mifsud, B. M. (2003). Victoria’s tallest trees. *Australian Forestry*, *66*(3), 197–205. https://doi.org/10.1080/00049158.2003.10674912

Milodowski, D. T., Coomes, D. A., Swinfield, T., Jucker, T., Riutta, T., Malhi, Y., Svátek, M., Kvasnica, J., Burslem, D. F. R. P., Ewers, R. M., Teh, Y. A., & Williams, M. (2021). The impact of logging on vertical canopy structure across a gradient of tropical forest degradation intensity in Borneo. *Journal of Applied Ecology*, *58*(8), 1764–1775. https://doi.org/10.1111/1365-2664.13895

Moncrieff, G. R., Lehmann, C. E. R., Schnitzler, J., Gambiza, J., Hiernaux, P., Ryan, C. M., Shackleton, C. M., Williams, R. J., & Higgins, S. I. (2014). Contrasting architecture of key African and Australian savanna tree taxa drives intercontinental structural divergence. *Global Ecology and Biogeography*, *23*, 1235–1244. https://doi.org/10.1111/geb.12205

Mora, F., Martínez-Ramos, M., Ibarra-Manríquez, G., Pérez-Jiménez, A., Trilleras, J., & Balvanera, P. (2015). Testing chronosequences through dynamic approaches: time and site effects on tropical dry forest succession. *Biotropica*, *47*(1), 38–48. https://doi.org/10.1111/btp.12187

Olson, D. M., Dinerstein, E., Wikramanayake, E. D., Burgess, N. D., Powell, G. V., Underwood, E. C., D’Amico, J. A., Itoua, I., Strand, H. E., Morrison, J. C., Loucks, C. J., Allnutt, T. F., Ricketts, T. H., Kura, Y., Lamoreux, J. F., Wettengel, W. W., Hedao, P., & Kassem, K. R. (2001). Terrestrial ecoregions of the world: a new map of life on earth. *BioScience*, *51*(11), 933–938. https://doi.org/10.1641/0006-3568(2001)051[0933:TEOTWA]2.0.CO;2

Paul, K. I., Roxburgh, S. H., Chave, J., England, J. R., Zerihun, A., Specht, A., Lewis, T., Bennett, L. T., Baker, T. G., Adams, M. A., Huxtable, D., Montagu, K. D., Falster, D. S., Feller, M., Sochacki, S., Ritson, P., Bastin, G., Bartle, J., Wildy, D., … Sinclair, J. (2016). Testing the generality of above-ground biomass allometry across plant functional types at the continent scale. *Global Change Biology*, *22*, 2106–2124. https://doi.org/10.1111/gcb.13201

Ploton, P., Barbier, N., Momo, S. T., Réjou-Méchain, M., Boyemba Bosela, F., Chuyong, G., Dauby, G., Droissart, V., Fayolle, A., Goodman, R. C., Henry, M., Kamdem, N. G., Katembo Mukirania, J., Kenfack, D., Libalah, M., Ngomanda, A., Rossi, V., Sonké, B., Texier, N., … Pélissier, R. (2016). Closing a gap in tropical forest biomass estimation: accounting for crown mass variation in pantropical allometries. *Biogeosciences*, *13*, 1571–1585. https://doi.org/10.5194/bgd-12-19711-2015

Poorter, L., Bongers, F., Sterck, F. J., & Wöll, H. (2003). Architecture of 53 rain forest tree species differing in adult stature and shade tolerance. *Ecology*, *84*(3), 602–608.

Poorter, L., Bongers, L., & Bongers, F. (2006). Architecture of 54 moist forest tree species: traits, trade-offs, and functional groups. *Ecology*, *87*(5), 1289–1301. https://doi.org/10.1890/07-0207.1

Schepaschenko, D., Shvidenko, A., Usoltsev, V., Lakyda, P., Luo, Y., Vasylyshyn, R., Lakyda, I., Myklush, Y., See, L., McCallum, I., Fritz, S., Kraxner, F., & Obersteiner, M. (2017). A dataset of forest biomass structure for Eurasia. *Scientific Data*, *4*(1), 1–11. https://doi.org/10.1038/sdata.2017.70

Schlund, M., von Poncet, F., Kuntz, S., Boehm, H. D. V., Hoekman, D. H., & Schmullius, C. (2016). TanDEM-X elevation model data for canopy height and aboveground biomass retrieval in a tropical peat swamp forest. *Https://Doi.Org/10.1080/01431161.2016.1226001*, *37*(21), 5021–5044. https://doi.org/10.1080/01431161.2016.1226001

Sellan, G., Simini, F., Maritan, A., Banavar, J. R., de Haulleville, T., Bauters, M., Doucet, J.-L., Beeckman, H., & Anfodillo, T. (2017). Testing a general approach to assess the degree of disturbance in tropical forests. *Journal of Vegetation Science*, *28*(3), 659–668. https://doi.org/10.1111/jvs.12512

Sellan, G., Thompson, J., Majalap, N., & Brearley, F. Q. (2019). Soil characteristics influence species composition and forest structure differentially among tree size classes in a Bornean heath forest. *Plant and Soil 2019 438:1*, *438*(1), 173–185. https://doi.org/10.1007/S11104-019-04000-5

Shenkin, A., Bentley, L. P., Oliveras, I., Salinas, N., Adu-Bredu, S., Marimon-Junior, B. H., Marimon, B. S., Peprah, T., Choque, E. L., Trujillo Rodriguez, L., Clemente Arenas, E. R., Adonteng, C., Seidu, J., Passos, F. B., Reis, S. M., Blonder, B., Silman, M., Enquist, B. J., Asner, G. P., & Malhi, Y. (2020). The influence of ecosystem and phylogeny on tropical tree crown size and shape. *Frontiers in Forests and Global Change*, *3*, 109. https://doi.org/10.3389/ffgc.2020.501757

Shenkin, A., Chandler, C. J., Boyd, D. S., Jackson, T., Disney, M., Majalap, N., Nilus, R., Foody, G., bin Jami, J., Reynolds, G., Wilkes, P., Cutler, M. E. J., van der Heijden, G. M. F., Burslem, D. F. R. P., Coomes, D. A., Bentley, L. P., & Malhi, Y. (2019). The world’s tallest tropical tree in three dimensions. *Frontiers in Forests and Global Change*, *2*, 32. https://doi.org/10.3389/ffgc.2019.00032

Sterck, F. J., Sterck, F. J., Bongers, F., Bongers, F., Newbery, D. M., & Newbery, D. M. (2001). Tree architecture in a Bornean lowland rain forest: intraspecic and interspecic patterns. *Plant Ecology*, *153*, 279–292.

Stillhard, J., Hobi, M., Hülsmann, L., Brang, P., Ginzler, C., Kabal, M., Nitzsche, J., Projer, G., Shparyk, Y., & Commarmot, B. (2019). Stand inventory data from the 10-ha forest research plot in Uholka: 15 yr of primeval beech forest development. *Ecology*, *100*(11), 2845. https://doi.org/10.1002/ecy.2845

Sullivan, M. J. P., Lewis, S. L., Hubau, W., Qie, L., Baker, T. R., Banin, L. F., Chave, J., Cuni‐Sanchez, A., Feldpausch, T. R., Lopez‐Gonzalez, G., Arets, E., Ashton, P., Bastin, J., Berry, N. J., Bogaert, J., Boot, R., Brearley, F. Q., Brienen, R., Burslem, D. F. R. P., … Phillips, O. L. (2018). Field methods for sampling tree height for tropical forest biomass estimation. *Methods in Ecology and Evolution*, *9*(5), 1179–1189. https://doi.org/10.1111/2041-210X.12962

Vovides, A. G., Berger, U., Grueters, U., Guevara, R., Pommerening, A., Lara-Domínguez, A. L., & López-Portillo, J. (2018). Change in drivers of mangrove crown displacement along a salinity stress gradient. *Functional Ecology*, *32*(12), 2753–2765. https://doi.org/10.1111/1365-2435.13218

Wirth, C., Schumacher, J., & Schulze, E.-D. (2004). Generic biomass functions for Norway spruce in Central Europe - a meta-analysis approach toward prediction and uncertainty estimation. *Tree Physiology*, *24*(2), 121–139.

Xu, Y., Franklin, S. B., Wang, Q., Shi, Z., Luo, Y., Lu, Z., Zhang, J., Qiao, X., & Jiang, M. (2015). Topographic and biotic factors determine forest biomass spatial distribution in a subtropical mountain moist forest. *Forest Ecology and Management*, *357*, 95–103. https://doi.org/10.1016/j.foreco.2015.08.010
